# Assistive technologies after stroke: self-management or fending for yourself? A focus group study

**DOI:** 10.1186/1472-6963-13-334

**Published:** 2013-08-22

**Authors:** Sara Demain, Jane Burridge, Caroline Ellis-Hill, Ann-Marie Hughes, Lucy Yardley, Lisa Tedesco-Triccas, Ian Swain

**Affiliations:** 1 Faculty of Health Sciences, University of Southampton, Southampton, UK; 2 School of Health and Social Care, Bournemouth University, Bournemouth, UK; 3 Electronics and Computer Sciences, Faculty of Physical & Applied Sciences, University of Southampton, Southampton, UK; 4 Psychology, Faculty of Social and Human Sciences, University of Southampton, Southampton, UK; 5 School of Design, Engineering and Computing, Bournemouth University, Bournemouth, UK; 6 Clinical Science and Engineering, Salisbury NHS Foundation Trust, Salisbury, UK

**Keywords:** Stroke, Upper limb, Assistive technology, Self-management, Patient, Family caregivers, Health care professional, Rehabilitation, Qualitative research

## Abstract

**Background:**

Assistive Technologies, defined as “electrical or mechanical devices designed to help people recover movement” have demonstrated clinical benefits in upper-limb stroke rehabilitation. Stroke services are becoming community-based and more reliant on self-management approaches. Assistive technologies could become important tools within self-management, however, in practice, few people currently use assistive technologies. This study investigated patients’, family caregivers and health professionals’ experiences and perceptions of stroke upper-limb rehabilitation and assistive technology use and identified the barriers and facilitators to their use in supporting stroke self-management.

**Methods:**

A three-day exhibition of assistive technologies was attended by 204 patients, family caregivers/friends and health professionals. Four focus groups were conducted with people purposively sampled from exhibition attendees. They included i) people with stroke who had used assistive technologies (n = 5), ii) people with stroke who had not used assistive technologies (n = 6), iii) family caregivers (n = 5) and iv) health professionals (n = 6). The audio-taped focus groups were facilitated by a moderator and observer. All participants were asked to discuss experiences, strengths, weaknesses, barriers and facilitators to using assistive technologies. Following transcription, data were analysed using thematic analysis.

**Results:**

All respondents thought assistive technologies had the potential to support self-management but that this opportunity was currently unrealised. All respondents considered assistive technologies could provide a home-based solution to the need for high intensity upper-limb rehabilitation. All stakeholders also reported significant barriers to assistive technology use, related to i) device design ii) access to assistive technology information and iii) access to assistive technology provision. The lack of and need for a coordinated system for assistive technology provision was apparent. A circular limitation of lack of evidence in clinical settings, lack of funded provision, lack of health professional knowledge about assistive technologies and confidence in prescribing them leading to lack of assistive technology service provision meant that often patients either received no assistive technologies or they and/or their family caregivers liaised directly with manufacturers without any independent expert advice.

**Conclusions:**

Considerable systemic barriers to realising the potential of assistive technologies in upper-limb stroke rehabilitation were reported. Attention needs to be paid to increasing evidence of assistive technology effectiveness and develop clinical service provision. Device manufacturers, researchers, health professionals, service funders and people with stroke and family caregivers need to work creatively and collaboratively to develop new funding models, improve device design and increase knowledge and training in assistive technology use.

## Background

Improved survival rates, increased prevalence of risk factors, better long-term management and an aging population are all predicted to contribute to the number of people living with the effect of stroke worldwide [1,2]. Eighty per cent of stroke survivors have upper limb impairments at stroke onset [3]. Estimates of recovery of useful function among severely affected patients vary widely depending on definition of ‘severe’ and ‘functional recovery’. Examples are 18% [4] and 11.6% [5]. It is generally agreed however, that loss of upper limb function impacts on quality of life of the stroke survivor and their family [6] as well as affecting ability to return to work. Stroke outcomes impact on national economies both through loss of employment and cost of health and social care [7,8].

Average inpatient stay following stroke is decreasing and is now 19 days in the UK [9] and 6.2 days in the US [10]. This means that increasingly rehabilitation is community-based and with reduced opportunities one-to-one intensive input for upper limb rehabilitation, which could affect recovery [11] (Kwakkel 2004). Stroke is now considered a long-term condition [12] and there is evidence in recent trials of technologies used to support physical recovery up to nine months post stroke, for example in the EXCITE trial of Constraint Induced Movement Therapy [13]. Despite this, support even for ‘self-managed’ rehabilitation beyond the first few months post-stroke is rare [14], and research evidence and policy decisions have not translated into practice.

Assistive Technologies, which we define as “electrical or mechanical devices designed to help people recover movement in their upper limb”, could be used to augment conventional therapy and address the challenges facing service providers. Assistive technologies may provide; increased intensity of therapy without a corresponding increase in clinical contact time, motivating relevant activities, either functional or impairment based, and can be used outside the hospital. They therefore have great potential to improve the cost effectiveness of upper limb stroke rehabilitation.

There is some evidence from clinical trials and systematic reviews to support the efficacy of a range of assistive technologies in upper limb stroke rehabilitation in reducing impairments and in some cases improving function including: robot therapy [15], Electrical Stimulation [16], Constraint Induced Movement Therapy [13] Virtual Reality [17] and non-invasive brain stimulation [18]. Rapid advances continue to be made in technology with commensurate investment both from the commercial sector and government funded research in The UK, Europe and the US. Consequently assistive technologies are increasingly used in clinical practice - often without established research evidence. Indeed, a recent review by Burridge and Hughes [19] identified that commercial drive is often a more important factor in their use than research evidence. It is likely that successful translation of assistive technologies into clinical practice will depend not only on robust, pragmatic research evidence but also on understanding translational aspects such as the influence of the type of service provision, practical aspects of device design and views of both service users and providers on the relevance and usefulness of assistive technologies.

This research forms part of a larger programme of research – the ATRAS project which covered four areas a) the current treatment methods and outcome measures used in England for upper limb rehabilitation following stroke b) systematic reviews of what assistive technologies exist to improve upper limb rehabilitation following stroke c) the current project, all of which would inform a clinical trial to assess the cost effectiveness of assistive technology combinations. The aim of this research was to identify current assistive technology knowledge and service provision and the barriers and opportunities for evidence based assistive technologies to be used in stroke upper limb rehabilitation practice, as perceived by stroke survivors, family caregivers and healthcare professionals.

## Methods

A qualitative design was used comprising an interactive exhibition and a series of four focus groups; two with patients, one with carers and one with health professionals. Ethical approval for the study was granted by the Isle of Wight, Portsmouth and South East Hampshire Research Ethics Committee (ref: 09/H0501/71) and consent was given by participants before data collection.

### Interactive exhibition

An interactive exhibition, with 12 different companies displaying 27 different upper limb Assistive technologies appropriate for stroke rehabilitation was held over 3 days at the University of Southampton. A range of stakeholders, including people with stroke, their friends, family members who provided care or support to a person with stroke (referred to as “family caregivers” throughout this paper, commissioners, budget holders and clinicians, and representatives from the local voluntary sector were invited. The aims of the exhibition were to increase stakeholder knowledge about assistive technologies, provide an opportunity for people to try and compare devices and to provide a useful means of recruiting relevant people to the study. Two hundred and four people attended (51% were people with stroke or family/friends and 49% were healthcare professionals). Delegates, many of whom spent three or four hours at the exhibition, were invited to ask questions and test each of the technologies presented to consider whether they addressed their needs. They were given a pamphlet illustrating the assistive technologies with space to add their comments, which they were invited to keep. Awareness was raised about the research study, and people were invited to leave contact and demographic details if they were willing to be contacted about taking part in a focus group (n = 31 health professionals; 32 patients and/or family caregivers).

### Focus groups

Potential participants who registered an interest in taking part in a focus group were purposively sampled in order to gather views from a range of stake-holders a) people with stroke of differing ages, genders, stroke severity, b) differing professional backgrounds and c) those who identified themselves as family caregivers. The number of focus groups was determined a priori, the main aim being to capture the breadth of opinions of the various stakeholders involved rather than to ensure data saturation for each particular stakeholder group. Invitations to attend focus groups on specified dates were sent to all those who responded. Those willing and able to take part on the dates specified (n = 21) were allocated to one of the four focus groups: Focus Group 1: people who had not used assistive technologies (n = 4 patients and 1 family caregiver); Focus Group 2: people who had used at least one assistive technology post-stroke (n = 4 patients and 2 family caregivers; Focus Group 3: family caregivers (n = 5; 1 of whom also attended focus group 1); Focus Group 4: Healthcare Professionals (n = 6). Inclusion criteria were a) stroke survivors, having a previous stroke at any time b) family care givers, who defined themselves as supporting a family member who had had a stroke and c) health professionals – engaged in stroke rehabilitation in the last 2 years. Although aphasia and cognitive impairments were not exclusion criteria, all participants were able to give informed consent. Focus groups were appropriate for exploring the complex and poorly understood issue of assistive technology use in stroke rehabilitation because they encourage questions and discussion, probe for depth, reflection on others’ opinions and reconsideration of personal views [20,21].

Each focus group was held at the University of Southampton and facilitated by a researcher (SD). An observer (AMH) noted non-verbal aspects of the group as is good practice when carrying out focus groups [22]. At the beginning of each focus group the participants were reminded that the researchers were interested in hearing their views on the use of technologies for helping people recover use of their arm and hand after stroke and reminded of the technology which was on show at the exhibition via individual information sheets and posters around the room. Categories of assistive technologies discussed were virtual reality (including commercially available gaming technologies such as the Nintendo Wii); dynamic splints; biofeedback; robots; constraint induced movement therapy and electrical stimulation. Four separate topic guides were created. The overall content and structure of the topic guide was similar for each group but modified slightly in address the needs of the participants. The topic guide for all groups can be seen in Table 1.

**Table 1:** Topic guide for the focus groups

	**Topics**
All Groups	Can I begin by asking what your overriding thoughts were about what you saw at the workshop?
	What role do you think assistive devices should play in the rehabilitation of the hand and arm? Early after stroke when people are still in hospital? In the first few weeks and months after people go home? What about longer after stroke – say one or more years later?
	Is there any other important information about the use of assistive technology in stroke rehabilitation that you think we should know?
Group 1	I have been told that most of you here had not tried any of these technologies before attending the workshop, is that correct? (If anyone has used assistive technology, we asked them to briefly explain what kind of technology they used and what they thought about it)
	For those of you who have not used assistive technologies, is this because you have never been offered them in the past? If not offered we asked why do they think you have not been offered it? If offered we asked Why did you decide not to use it?
Group 2	As a group of people who have used technologies to help with your arm rehabilitation what do you think are the main advantages to using such devices? What about the main disadvantages?
	Would some of you mind telling me about how you came to use an assistive technology?
	If we could wave a magic wand and offer you any of the assistive technology devices you saw at the workshop which would it be and why?
	Were there any devices you would not be keen to use and why?
Group 3	What do you think are the main advantages of using assistive technology for the arm?
	Do you think there are any disadvantages of using assistive technology for the arm?
	Why do you think assistive devices are not used more in arm treatment?
	What opportunities do you see for using assistive technologies in arm treatment in the future
	If you could wave a magic wand and select one assistive technology device for your relative to use, which would it be and why?
	Which do you think would be least useful and why?
Group 4	What do you think are the main opportunities or advantages of using assistive technology in upper limb rehab?
	Do you think there are any disadvantages to using assistive technology with the arm?
	Which patients do you think assistive technologies are most useful for?
	Are there any patients you would choose not to use assistive technologies with?
	Why do you think assistive devices are not used more in upper limb? What opportunities do you see for using assistive technology in upper limb rehab in the future rehabilitation?
	If you could wave a magic wand and select one assistive technology which you think would be appropriate for your patients and your service which would it be and why? Which do you think would be least useful and why?

Audio-recordings of each group were transcribed verbatim; observed and noted non-verbal information was incorporated into the transcripts. Pseudonyms were allocated to maintain confidentiality. Data were managed using NVivo 8 software (QSR International) and analysed thematically [23].

Transcripts were read, re-read and coded inductively by SD and AMH, cognisant of the research questions. For instance the quote from the family care giver focus group *“we applied for funding and were turned down”* was coded as ‘funding application refused’. SD and AMH discussed and grouped codes with related meaning to generate emergent themes for each focus group: for instance ‘funding application refused’ was grouped with ‘purchasing private physiotherapy’ to form the family focus group theme of ‘financial impact on families’. Themes generated for each focus group were then compared and contrasted across all focus groups to highlight similarities and differences in the data and generate the final overarching thematic structure presented in the findings; for instance ‘financial impact for families’ (family caregivers focus group) was combined with ‘complex commissioning process’ (health professional focus group) under the final overarching theme of ‘access to assistive technologies. Codes and developing themes were discussed with the wider research team throughout this process to highlight alternative interpretations and agree analytical decisions.

## Results

Demographics of participants are provided in Table 2. There were more women caregivers and more men with stroke. Four of the family caregivers were partners of stroke survivor participants. All stroke survivor participants had experienced upper limb impairment post-stroke of whom three had recovered functional use. Four patients had used assistive technologies (robots, electrical stimulation and dynamic splints) before the exhibition, none had used virtual reality which was the most commonly used assistive technology amongst the health professional group. Conversely none of the health professional group had experience with robot therapy.

**Table 2:** Demographic details of the patient, family caregivers and health care professional cohorts

	**Patient (AT naïve and experienced)**	**Family caregivers (AT naïve and experienced)**	**Health care professionals**
	**n = 8**	**n = 7**	**n = 6**
**Age (years)**	46-79	44-82	Not applicable
**Gender**	1 female	6 female	5 female
	7 male	1 male	1 male
**Living situation**	2 alone	1 alone	Not applicable
	6 with partner/spouse	6 partner/spouses	
**Time since stroke (years)**	1-12	2-13	Not applicable
**Employment status**	1 working	1 working	Qualified 6–24 years
**Experience of using AT prior to exhibition**	4 none	4 none	1 none
	4 prior experience	3 prior experience	5 prior experience
**What ATs used before exhibition**	1 Robot	1 Robot	3 ES
	2 ES	1 ES	4 Dynamic Splints
	1 Implanted ES	1 Implanted ES	4 Virtual reality
	1 Dynamic Splints	1 Dynamic Splints	/Computer games
**Professional status**	Not asked	Not asked	2 Physiotherapist (NHS) 1 Physiotherapist (Private practice), 1 Occupational Therapist (NHS), 1 Occupational Therapist (Social Services), 1 Clinical Registrar

*AT* Assistive technology, *ES* Electrical stimulation.

The qualitative thematic analysis identified the potential of assistive technologies to support self-management in stroke rehabilitation as a core concept in all the focus groups. Three themes were also generated a) device design, b) access to information about assistive technologies, and c) access to using assistive technologies. Each of these themes impacted on stakeholders’ opportunity to self-manage and experiences of and views on assistive technology use and provision. Pseudonyms (fictitious names) are used throughout.

### Assistive technologies as a tool for supporting self-management in stroke rehabilitation

The potential of assistive technologies to facilitate self-management was a core concept running through each of the focus groups.

A focus on mobility and limited attention to arm rehabilitation post-stroke has been noted previously [24]. The patients and family caregivers in this study, similarly reported a therapeutic emphasis on gait and mobility whilst in hospital. They had assumed that once home, arm and hand rehabilitation would take a greater priority, but for most people this was not the case. They also reported a discontinuity between therapy in hospital and at home, with long waits before home-based therapy commenced and a reduction in the intensity when it did. This was worrying for them. Many participants had a lay understanding of the principles of neuroplasticity and that intensity and repetition were necessary to optimise functional recovery.

*They concentrate very hard on getting you walking again, which, quite right, it’s a priority, but after all them getting you walking again, there’s very little on, on the arm, in our experience.* [Marie; family caregiver; no prior assistive technology use; focus group 1]

*(It’s important to keep) brain cells, and nerve endings, connected in some way, just keep it going. I think the minute it all stops, it’s just wasted time in the hospital… you know, you come out of hospital, and they, they say they’ll give you an hours Physio a week!* [Sheila; family caregiver; prior assistive technology use; focus group 3]

Assistive technologies were suggested as a solution to this disconnect. People with stroke and their families suggested that they could be taught how to apply and use assistive technologies whilst in hospital, be provided with an assistive technology to take home and then use this to deliver intense, repetitive therapy both before and after their home therapy commenced.

*I think that it (assistive technology use) has got to start before you are, before you are discharged, to be able to carry it home, and then do whatever it is you need to do afterwards.* [Susan; family caregiver; prior assistive technology use; focus group 3]

The health professionals were more ambivalent about using assistive technologies to facilitate the transition home. The hospital-based therapists were concerned about how they would find the time to prescribe and teach people how to use assistive technologies, given the focus on facilitating discharge.

*the difficulty is there’s such a drive to get patients out of hospital so early…you’re spending a lot of your time getting them ready to be at home, so you don’t have time.. I think that’s a real challenge of how you start introducing assistive technologies.* [Maggie; OT; prior assistive technology use; focus group 4]

But they recognised the potential for assistive technologies to provide intensive therapy and a means of self-management.

*I think in terms of evidence then repetition, repetition, repetition, exercise, exercise, exercise is hugely important… which is what we, as staff can’t give them.* [Sarah; physiotherapist (private practice); prior assistive technology use; focus group 4]

[assistive technology provision] *is also preparing people at the time when they go home … that they’re in charge of it, you know, that they have a big role to play in their rehabilitation. *[Amy; occupational therapist; prior assistive technology use; focus group 4]

All patient participants were keen to self-manage. They were all actively engaged in looking for solutions to promote arm recovery and were prepared to spend time and, if necessary, money on potential solutions, including assistive technologies as highlighted by Keith:

*(I want) something I could use every day….(not) just go to a (physio) centre once a week or so and by the time the six days has gone past not using it you might be back where you were before.* [Simon; person with stroke; no prior assistive technology use; focus group 1]

The opportunity for self-management was influenced by a) device design b) access to information and access to devices.

### Device design for self-management

If assistive technologies are to support self–management they need to be simple to apply, easy to use, motivating and to provide feedback on performance.

Patients, family caregivers and health professionals all recognised the motivational aspect of assistive technologies. They were seen as an improvement on routine therapy; the fact that they were ‘hi-tech’ and designed specifically for rehabilitation made them more credible and enjoyable than traditional therapy exercises which were often deemed to be boring and difficult to notice improvement:

*He would have spent hours on your robot because it has flashing lights; it was connected to an electricity source, and it looked hi-tech (laughter). He would die before he will sit at home at the table and do this (polish table) for 10 minutes at a time, because, well, “it’s stupid, it’s pointless”. And, and I think having a piece of equipment makes, it also makes you feel you are actually doing Physio.* [Sheila; family caregivers; prior assistive technology use; focus group 3]

*it’s a visual reminder, it’s a real … I got a girl to buy, she had a, a Wii and she got the Guitar Hero, and my goodness, (laughter) the improvement in her (yeah) finger activity that helped her typing, but she didn’t want to practice typing because she had to do that for university study, but by using, you know, something that is enjoyable and she could see, get some really good visual feedback, Wow! The recovery she got was absolutely fantastic.* [Maggie; occupational therapist; prior assistive technology use; focus group 4]

The preference for technological solutions seemed to be particularly true of the younger, male patients in our study; a view reiterated by the therapists, who also suggested that patient acceptance of, and demand for, technologies would increase as the technical competency of the population improved.

*in the longer term 20 – 50 years down the line everyone’s going to know what computers are (yeah) and technology is, so , so you’ve got to look at that , the inter-activeness, and what’s on our computer screen is going to become very important. And it has to be goal orientated, like your guitar hero … So the simplistic things [routine therapy], although they work and they’re cheaper, they’re still not goal orientated enough , I don’t think, for the clients to repeat it enough to get the change and the plasticity that we’re looking for.* [Sarah; physiotherapist (private practice); prior assistive technology use; focus group 4]

The time taken to prepare, set up and maintain assistive technology devices was seen as a key issue for all stakeholders. For therapists, the devices were viewed as a tool for improving productivity and effectiveness by enabling more patient practice hours per therapist. Concerns were expressed about devices which needed complex adjustment between patients (robots and dynamic splints), which might be difficult to move to the patient (robots), which were complex to programme (ES, robots), which were time consuming to clean (most products) and difficult to store (robots in particular). For patients and families, the devices needed to be easy to get on and off a weak and/or contracted hand/arm (problems identified with splints and some robots) and to be intuitive in terms of correctly positioning the device (problems identified with some electrical stimulation devices and robots).

*electrical stimulation is a lot of bother. I mean Pete, it took Pete a long time before he was confident of putting it on himself; he would always call me, “Is it in the right place? Will you do it?”* [Sheila; family caregivers; prior assistive technology use; focus group 3]

*The splint (Saeboflex) is very fiddly and you know, he (company representative) couldn’t even put it on my hand … I can’t imagine if it would have to be done every day*. [Patricia; person with stroke; no prior assistive technology use; focus group 1]

*Make them (robots) much more user-friendly. I think they are such big bits of kit. You can imagine, it’s like taking an X-ray machine onto a ward…We’ve only got in a day, 20 minutes, twice, to work on a limb. I prefer to give them exercises and go, “just keep working, keep working”.* [Liz; physiotherapist; prior assistive technology use; focus group 4]

Good device design is critical for self-management. This research indicates that there is scope to improve the design of currently available assistive technologies It is clear that therapists, people with stroke and their families can all provide useful feedback on and suggestions for improvements in design.

### Access to information

Many of the patients had not known anything about assistive technologies prior to attending the exhibition. Several expressed amazement at what they had seen and tried out and wished there were more similar opportunities available. Other participants had used assistive technologies for their leg but not been offered anything for their arm, whilst others had used assistive technologies for their arm whilst in hospital but had had to return the devices on leaving hospital.

*I was supposed to find that it (electrical stimulation) would improve my hand, which it did. So I was disappointed when it was taken away. And I said that I was not happy with it being taken away, and it was still taken away anyway.* [Paul; person with stroke; prior assistive technology use; focus group 2]

Several patient and family caregiver participants had sought out information on assistive technologies. This generally occurred after the people were discharged from therapy but were still looking for a solution to their persistent arm and hand disability. They gathered information from a variety of sources; other people affected by stroke who used assistive technologies, information in the national press, the internet and from sales representatives. Patient and family caregivers worried about the quality of the information available from these sources and the relevance of the information to their own situation. They would have liked to be able to seek advice from a therapist they knew and trusted.

Participants in each group suggested that they had not been given more information on assistive technologies by therapists, because a) therapists were overworked, b) lacked knowledge and training about what was available, and c) were reluctant to give information about devices that they could not provide within the state funded service.

*I think their (health professionals’) time is very constrained anyway, and that’s why they have this problem with actually sort of using new equipment. That’s my personal opinion. And it’s funding. It’s the biggest issue of all. We (patients) might know what we want; we know what we’d like (yep, yep, yep), it’s actually getting it, you know. And all right, some people can fund it themselves, but they still need to be able to get to the right people to actually give them that equipment…(its knowing) what you can and can’t get…it’s a matter of education.* [Martin; person with stroke; prior assistive technology use; focus group 2]

*it’s not showing us the things…you get such a very, very negative… I mean we have had one or two fabulous Physios, but if they aren’t enthusiastic about whatever technologies you’ve researched and discovered work, and if they’re not given the support and the time to use them, then actually all the research on how good they are just falls into a big black hole…. It’s the complete mind-set. You know, the Physios were so disheartened. I think the Physios tend very much to say “No it won’t work” because they don’t want you thinking that there’s a miracle cure that you can’t afford. So they’ll always try and close down your options, because then you’re not demanding things.* [Sheila; family caregivers; prior assistive technology use; focus group 3]

Interestingly one of the therapists confirmed these opinions when she expressed her concerns over the time pressures assistive technology prescription could generate for her service:

*I think there’s a number of patients that it would be useful for but I think the cost versus the, not just the cost financially, but the cost that you’d have to have people coming in begging me for it.* [Liz; physiotherapist; prior assistive technology use; focus group 4]

The patient and family caregiver participants felt strongly that health professionals should give them access to information so that they could choose whether or not to purchase equipment for themselves, as this discussion demonstrates:

[Discussion in focus group 3 between Sheila and Louis, two family caregivers]

Sheila: nobody actually makes you aware

Louise: or answers the questions

Sheila: of the things that, you know, you could choose. Or if you didn’t want to, you could choose not to follow up. Because I think if the information’s given to you, you would at least have a choice… Um, so more advice and, and opportunity to see these things would be tremendously helpful, because you could actually make a balanced judgment.

As this was an issue over which patients and family caregivers had expressed strong feelings we sought out health professionals’ views about the level of information provided. Many dilemmas were apparent including a) the lack of strong research evidence for upper limb assistive technologies, b) concerns over inequity in assistive technology provision and c) tensions about highlighting the existence of a device which may help but which is not available from state-funded services. An interesting contrast between the views of those working in the state-funded system and the therapist working in private practice was noted. Both the private and state-funded physiotherapists agreed that the evidence base underpinning the use of assistive technologies in stroke arm rehabilitation was weak. Those working in the state system said this made them reluctant to talk to patients about assistive technologies in case it influenced them or their families to purchase something that may not work. In comparison, the private practice therapist suggested that if a patient asked about a device they had seen on the internet she would help them establish what the evidence base was and, if they wanted, arrange a meeting with the company representative. She indicated that many patients and families were accessing this information on the Internet anyway and that they needed therapists’ support in making an informed decision. However, both the state and privately funded therapists indicated that, whilst they would respond to a patient’s request for assistive technology information they would not proactively inform about devices, for fear of creating the impression of endorsing a product for which there was insufficient evidence, as this discussion indicates:

[Discussion in focus group4 between Amy (occupational therapist working in state funded National Health Service) and Sarah (physiotherapist working in private practice)]

Amy - There’s a bit of a moral dilemma there because we’ve talked about there not being a lot of good evidence for a lot of these things and how the NHS (state-funded health service) can’t fund them. Personally I would have a real issue with saying to someone, “There is this out there; it may or may not work”, knowing that they’re in a position in their life where they’re very emotionally heightened, and may not be thinking the most rationally they’ve ever done; they might also have cognitive impairment; even their relatives might not be, you know, they’re in, they’re in a state of shock or panic or whatever, and to think that I might influence how someone goes and spends their money, without good evidence.

Sarah: But, that’s exactly what you need to say to the patient.

Amy: I’d have a bit of a moral dilemma with that.

Sarah: Because everyone at some point is active with the internet, but again when they’re in the acute setting, they’re not exactly, you know, on the computer, but their relatives are.… I mean, my patients come to me… I don’t know how many times, they’ve Googled something new, and I’ll say, “Fine, if you want to go and have a look at it, you need to look at the evidence. I will not suggest this, but it is out there.” So I think for the clients now, they are so much more worldly wise, and we, we, I think I struggle to keep up ….I think it is, it is up to us to stand up and say, “Look, the evidence isn’t there, we as therapists cannot say that you need to go look at it, but if you’d like to I will contact the company and set up a trial for you.”

Health professionals were not only concerned about a lack of evidence for benefit they were also worried about the potential risk of harm, especially if they were to give advice about a device but could not provide adequate follow-up to ensure its safe use in the community.

*If I can’t ensure that that follow-up is going to be there, then I won’t provide it (assistive technology) to them because it is potentially more detrimental for them to just have it without a follow-up than for me not to give it to them.* [Lisa; physiotherapist; prior assistive technology use; focus group 4]

HPCs’ concerns extended beyond the risks to the patients and included risks of litigation to themselves and their organisations:

*In the age that we live in, you know with litigation and what have you [laughs], it’s quite scary to think that someone, you know, would misconstrue something you might say as recommending that thing, but then that doesn’t help them or does them harm.* [Amy; occupational therapist; prior assistive technology use; focus group 4]

In contrast, some of the patients and family caregivers were more willing to accept risks. In the absence of health service provision, one couple had purchased their own assistive technology device (electrical stimulation) on-line. To do this, however, the patient’s wife (Mary) had had to pretend that she was a health professional. The sentiments expressed indicate that Mary thought it safe for her and her husband to use the electrical stimulation because they had already been shown how to use it, could follow the instruction booklet and her husband could feel whether he was getting the right dose.

[Discussion between in focus group 3, between Mary and Susan, two family caregivers:]

Mary: I just sort of bought a (electrical stimulation) machine and carried on, because I couldn’t believe that you could see it was doing some good, and then you finish your re-hab time in hospital, and guess what? It’s gone … So while we were in the rehab hospital, I read the label on their machine, and I phoned that company up, and she said, “Are you a Physio?” And I said, “Sort of” (laughter). I got one anyway.

Susan: Yes, and how did you get to know how to use it?

Mary: because there’s an instruction booklet (OK), and, he himself would know if it was too strong, because of the toning, he could feel it. You know they get like a tone after a stroke. They know how much they can take and I would put it on very, very low.

Susan: How do you know where to put the sensors though?

Mary: It tells you in the booklet.

Mary’s comments suggest that she considered her and her husband to have sufficient expertise to use the electrical stimulation. The questions from Susan indicated a more cautious approach than Mary, but interestingly, after the focus group, Susan asked Mary for details of the electrical stimulation company with a view to purchasing one for her husband.

A similar view of expertise was also expressed by one of the patients. He suggested that patients could train health professionals how to use certain assistive technologies because they (the patients) may be more familiar with the devices than the therapists or nurses.

*I think probably with the SaeboFlex, if you use it for three of four times, if you get somebody new, like a new Physio come in, you could tell her (laughs) how to set it* [Dan; person with stroke; prior assistive technology use; focus group 2]

The health professionals were also concerned about the risk of giving false hope of recovery. In the absence of clear evidence about the effectiveness of assistive technologies, they worried that prescribing or giving information about such devices would generate unrealistic expectations which would be harmful for patients, and difficult for therapists to manage.

[Discussion in focus group 4 between Liz (physiotherapist working in National Health Service) and Sarah (physiotherapist working in private practice]

Liz: But you have to be careful that we’re not giving people some unrealistic expectation…hopefully people will get better recovery from this, but my concern is, is managing expectations which is quite hard.

Sarah: It’s also offering the patient false hope … you’re, you’re building them up to a degree that it’s definitely not going to solve. And patients do tend to hop from one technology to the other, … it does become ridiculous, because you get people coming out and showing you this wonderful fandangled thing that’s not very useful.

The patients and family caregivers were aware of health professionals’ reluctance to raise hopes and of the arguments about lack of evidence. However, they were less interested in generic findings, arguing that every person with stroke is different and that evidence of benefit should be sought on a case-by-case basis.

*But it’s not just the fact of getting other people’s hopes up when it’s an individual thing; because each one of them will progress in a different way, and what helps one won’t help another. So you can’t generalise a stroke person I don’t think.* [Mary; family caregivers; prior assistive technology use; focus group 3]

One patient and his wife, who reported gaining benefits from a trial of an upper-limb assistive technology, were frustrated that the commissioners based their funding decisions on generic evidence rather than on what they believed had already been proven to ‘work’ for them. This leads us to consider the provision of assistive technologies and the implications for this on self-management.

### Accessing equipment

A recurrent theme in all of the focus groups was the lack of funding for upper limb assistive technologies. This hampered the potential of assistive technologies to facilitate self-management.

From the health professionals’ perspective the lack of robust research evidence for upper limb assistive technology use was the biggest barrier to assistive technology provision. They recognised that clinical guidelines did not yet recommend assistive technologies for routine upper-limb rehabilitation, that much of the research was not independent of the companies that endorsed the products and that commissioners were faced with competing demands on their budgets.

*I took the upper limb electrical stimulation to the priority committee and they discussed it at the same time as lower limb (electrical stimulation). “Low priority” [they said] “because the research isn’t solid to back that up”. So that's going to be a real challenge to go back to the commissioners and say, “Oh, commission this for stroke services, you know, there might be some benefit”. And they’re going to look and go…actually that compared to this drug that we know, if you give this drug three times a day we’re going to put some heart condition, that’s going to make a, a benefit compared to something that hasn’t got good research.* [Liz; physiotherapist; prior assistive technology use; focus group 4]

On the other hand, the health professionals were also keen to explore the effectiveness of assistive technologies with individual patients. One therapist had organised a training course to raise money for some dynamic splints. She was hopeful that, if she could demonstrate individual benefit, the orthotic department would be persuaded to provide them for individual patients. Other therapists indicated they had similar schemes to “trial” assistive technologies with individuals, but that, even if these trials indicated benefit, getting funding for individual devices could not be assumed.

People with stroke and their family caregivers focussed more on lack of funding rather than lack of evidence as the reason why assistive technologies were not available. Two people in our study had used an upper limb assistive technology as part of their state-funded rehabilitation. In both cases this was a temporary loan, with assistive technology use ending due to hospital discharge rather than, they believed, on the basis of a clinical rationale. The patients and families typically believed that if a device was demonstrated to ‘work for them’ that was sufficient evidence that is should be provided for them, regardless of transitions between hospitals or services.

Many of the patients and family caregivers also discussed assistive technology cost as a barrier to them self-funding. Some indicated that they would be prepared to self-fund devices if they were able to test them first for personal benefit. This, they indicated needed to be more than a single trial with a company representative; they wanted to use the device for a period of time to see if they could detect benefits. As far as the patients and families were concerned generic evidence was neither necessary nor sufficient; they wanted to know it worked for them in their daily lives:

*if there was a way that you could have a bit of a trial … then I think you, you might try and sort of scrimp and save for, and try and get hold of one, but you can’t do it on a whim.* [Sheila; family caregivers; prior assistive technology use; focus group]

## Discussion

The findings as a whole indicate that these patients, family caregivers and health professionals with an interest in this area were all hopeful about the potential of assistive technologies to facilitate self-management and improve upper-limb recovery in stroke. The need to increase intensity and repetition of upper limb exercise by facilitating independent practice was recognised by all. Assistive technologies were seen as a potential solution to this.

Before focusing on the implications of these findings, it is important to consider the framework of this research. This research was conducted with people who had expressed an interest in the use of upper-limb assistive technologies in stroke-rehabilitation by attending an exhibition of assistive technology devices. They should, therefore, not be considered representative of the whole population affected by and working with stroke. By attending the exhibition and the focus groups our participants are likely to have more knowledge of and be more positive about the potential of assistive technologies than the population as a whole. We did, however, seek to include the views of a range of stakeholders that included people with stroke and family care-givers with variations in gender and age, who had experience of assistive technologies as well as those who were naïve prior to the exhibition. Health professionals were from the state and private sectors and had a range of assistive technology experience. The strength of this purposively selected group is that they were able to give a depth of views from a range of perspectives. It would be useful to carry out a wider survey of the issues to explore the issues identified further and this is underway.

There are two main issues raised by our findings, each of which will be discussed in turn: i) the urgent need for evidence from clinical settings to support assistive technology use in stroke upper-limb rehabilitation and ii) other potential barriers to the use of assistive technologies in the self-management of stroke.

From the patients’ and family caregivers’ perspective, little therapy is currently being provided for their arms or hands; assistive technologies offer the hope of improvement, but they are unable to access unbiased professional advice and risk buying products that may not meet their needs. The extent of the effectiveness of different assistive technologies has not been established in clinical settings. Therapists are reluctant to provide advice on assistive technologies, have difficulty assessing the impact of assistive technologies on their practice, and cannot gain funding for assistive technologies for their patients.

The prevalence of people living with stroke related disability is likely to increase as the population ages. Gathering evidence for therapies which can be both intense and used without therapist supervision, e.g. assistive technologies would be a useful research priority for governments and research funders. Assistive technology use is often underpinned by sound theoretical and evidence based principles at ‘experimental’ level. However translational work to everyday practice is needed which can take into account the heterogeneous nature of the stroke population, exploring the possible differences in benefits for different groups (i.e. large/small infarcts, presence/absence of somatosensory deficit, cognitive impairment etc.).

As well as the issue of research evidence, our findings highlighted several issues associated with self-management practice. The HPCs in this study felt that that many people struggled to become active managers of their own rehabilitation. However, the patients and family caregivers described how they actively sought solutions to their on-going upper-limb disability including searching for information on the internet, contacting companies, and purchasing their own assistive technologies. Kielmann et al. [25], similarly identified people with respiratory illnesses who saw themselves as active self-carers but who, like the participants in our study, complained of obstacles within the health system which acted as barriers to their self-management and who felt abandoned by the health service. Kennedy et al., [26] suggest that self-management cannot focus only on educating patients how to self-care and passing all responsibility for self-management to them. They suggest that self-management requires a whole systems approach which trains health professionals how to work in partnership with patients, and which encourages self-referral to services when patients need advice. Such a partnership approach would be vital to more routine use of assistive technologies in clinical practice.

Ensuring individuals can make informed choices and are supported to use technology are considered key to promoting self-management [24]. In contrast, the patients and families in this study were frustrated that they were not provided with information on assistive technologies by experts who they could trust, realising that a lack of expert advice increased the chance of them wasting their money or purchasing a device which might do harm. Lack of appropriate information is a recurring theme in audits of stroke services [14,25]. Despite their frustrations, the patients and families did not attribute the problem to individual therapists, who they viewed as overwhelmed and over worked; a barrier that professionals have recognised to their engagement with self-management working [27]. Research suggests that many health professionals have received little formal training to develop self-management skills [28]. Stroke specific training in self-management for staff working in stroke rehabilitation has recently become available [29] but is still undergoing development and evaluation.

A further concern, expressed by all stakeholders in this study, was the usability of the devices; an issue which has previously been reported as a barrier to self-management by health professionals [30]. Our findings identified design issues with the devices themselves which, if resolved, may encourage the adoption of assistive technologies into upper-limb rehabilitation. The use of an iterative design process, which embeds stakeholder feedback throughout the design cycle, is advocated [30]. Simply improving the devices is not, however, likely to be enough. Other psychosocial and organisational factors including communication channels, time, and the social system will also impact on device uptake [31]. Moving from adoption to the imbedding of technologies into routine practice is also problematic. Normalisation Process Theory [32] offers propositions to explain some of the integration issues identified in this research. For instance, the problem of ‘raising false hopes’ may hamper the integration of assistive technologies into practice because of the interactional challenge generated between professionals and patients; whilst the systemic problems relate to issues about the allocation and control of resources and how assistive technology provision can be integrated into existing patterns of activity. Both of these factors pose challenges to the normalisation of assistive technologies in rehabilitation practice that will need to be addressed if they are to serve as useful tools for self-management.

## Conclusions

Assistive technologies are viewed as promising tools for self-management in the rehabilitation of the upper limb by patients, families and professionals but there are significant barriers to their adoption and routine implementation in practice. Most self-management interventions focus on changing patient behaviour to encourage self-management, but this was not found to be the most critical issue in this study. The patients and families we spoke to demonstrated many key self-management motivations and behaviours. This research identified the devices, the evidence base, patient-professional relationships and the health system as the key barriers to the use of assistive technologies in upper-limb rehabilitation. Assistive Technologies should be co-designed by all stakeholders, and, given the aging population and the drive for self-management approaches, there is an urgent need to test their effectiveness in pragmatic clinical trials, rather than under ideal trial conditions, to expedite integration of effective assistive technologies into routine practice. A whole systems approach will be required which considers training for professionals and system modification if the promise of assistive technologies is to be realised.

## Competing interests

Jane Burridge – Scientific Advisory Board Hocoma A/T, previous consultancy for Ottobock and Bioness. Ian Swain - Director and shareholder in Odstock Medical. All other authors have no competing interests.

## Authors’ contributions

SD contributed to the design of the study, undertook acquisition and analysis of data and led on the writing of the paper. IDS was the Chief Investigator for the whole ATRAS project and commented upon the progress and final draft of the manuscript. JB made substantial contribution to conception, design, and interpretation of data and contributed to the writing of the paper. CEH participated in the design of the study, assisted with the analysis and helped to draft the manuscript. AMH undertook acquisition and analysis of data, as well as revising the manuscript. LTT assisted with the analysis of the data and read and approved the final manuscript. LY commented on the study design and analysis. All authors read and approved the final manuscript.

## Pre-publication history

The pre-publication history for this paper can be accessed here:

http://www.biomedcentral.com/1472-6963/13/334/prepub
